# New insights into iNKT cells and their roles in liver diseases

**DOI:** 10.3389/fimmu.2022.1035950

**Published:** 2022-10-26

**Authors:** Xinyu Gu, Qingfei Chu, Xiao Ma, Jing Wang, Chao Chen, Jun Guan, Yanli Ren, Shanshan Wu, Haihong Zhu

**Affiliations:** ^1^ State Key Laboratory for Diagnosis and Treatment of Infectious Diseases, National Clinical Research Center for Infectious Diseases, National Medical Center for Infectious Diseases, Collaborative Innovation Center for Diagnosis and Treatment of Infectious Diseases, The First Affiliated Hospital, Zhejiang University School of Medicine, Hangzhou, China; ^2^ Zhejiang University School of Medicine, Hangzhou, Zhejiang, China

**Keywords:** NKT cells, cytokine, chemokine, liver diseases, immune

## Abstract

Natural killer T cells (NKTs) are an important part of the immune system. Since their discovery in the 1990s, researchers have gained deeper insights into the physiology and functions of these cells in many liver diseases. NKT cells are divided into two subsets, type I and type II. Type I NKT cells are also named iNKT cells as they express a semi-invariant T cell-receptor (TCR) α chain. As part of the innate immune system, hepatic iNKT cells interact with hepatocytes, macrophages (Kupffer cells), T cells, and dendritic cells through direct cell-to-cell contact and cytokine secretion, bridging the innate and adaptive immune systems. A better understanding of hepatic iNKT cells is necessary for finding new methods of treating liver disease including autoimmune liver diseases, alcoholic liver diseases (ALDs), non-alcoholic fatty liver diseases (NAFLDs), and liver tumors. Here we summarize how iNKT cells are activated, how they interact with other cells, and how they function in the presence of liver disease.

## Introduction

Natural killer T (NKT) cells are a group of innate immune cells first recognized in the 1990s (1). These cells feature surface receptors of both T cells and NK cells (e.g., NK1.1 in mice or CD161+/CD56+ in humans). The activation and deactivation of NKT cells are closely tied to our immune activities, such as pathogen elimination, tumor surveillance, and autoimmune responses (2–4). NKT cells can be divided into two subtypes, namely type I and type II. Type I NKT cells, usually referred to as invariant NKT (iNKT) cells, express a semi-invariant mouse Vα14-Jα18/Vβ8 or human Vα24-Jα18/Vβ11 T cell-receptor (TCR) α chain, hence the name. Type I NKT cells are able to recognize lipid antigens (such asglycosphingolipids, glycerophospholipids, lysophospholipids, and cholesterol ester) in the context of CD1d, a non-polymorphic MHC class I-like molecule (5–8). Researchers have found that the injection of α-galactosylceramide(α-GalCer) activates type I NKT cells (9). Type II NKT cells, in contrast, express a relatively diverse range of TCR receptors, and are reactive to a self-glycolipid sulfatide (10). Studies preliminarily suggest contradictory functions for the two types of NKT cells: type I NKT cells are likely pro-inflammatory, while type II are anti-inflammatory (6, 11). Note that type II NKT cells have not been broadly studied due to a lack of distinctive surface characteristics. In this review, we mainly focus on iNKT cells, with also a few contents talking about type II NKT cells, and “NKT cells” will stand for iNKT cells unless otherwise stated.

The liver is a vital part of the human digestive system, and functions as the center of metabolism and detoxification. Though not seen as a primary immune organ, the liver is not to be neglected when we talk about immune reactions. In addition to parenchymal cells (i.e., hepatocytes), the liver also hosts non-parenchymal cells, such as liver sinusoidal endothelial cells, Kupffer cells (macrophages), lymphocytes, and stellate cells. Interestingly, the liver has the highest NKT cell/conventional T cell ratio in the body (12), suggesting that this organ might play an important role in NKT immunology. In this review, we mainly focus on two points: 1) how NKT cells are activated, and 2) how NKT cells interact with other cells (e.g. Kupffer cells, T cells, hepatocytes) in the presence of liver disease.

## NKT-cell activators

**Figure 1:**
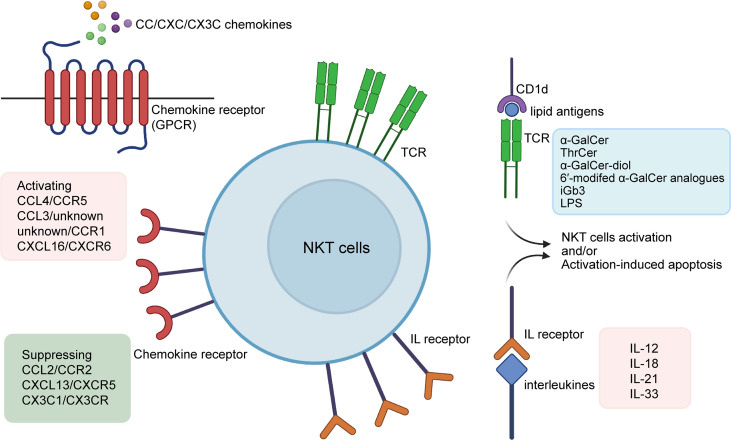
Activators of NKT cells. α-Galcer and some of its analogues (e.g.ThrCer, α-GalCer-diol, 6′-modifed α-GalCer analogues), iGb3, and LPS are among lipid antigens proven to activate NKT cells *via* the CD1d-dependent pathway; interleukins such as IL-2, IL-12, IL-15, IL-18, IL-21, and IL-33 promote NKT cells by binding directly to their interleukin receptors; CC/CXC/CX3C chemokines are associated with the recruitment and proliferation of NKT cells, with non-exclusive matches with their receptors that come in the form of GPCRs. Also, α-Galcer and some interleukins may also lead to activation-induced apoptosis of NKT cells. α-GalCer, α-galactosylceramide; ThrCer, threitolceramide; iGb3, isoglobotrihexosylceramide; LPS, lipopolysaccharide; IL, interleukin.

Briefly, it is known that NKT cells can be activated by lipid antigens (especially α-GalCer) (13–15) cytokines (such as IL-2 (interleukin-2) and IL-18) (16–18) chemokines (including CXCR6) (19), and other substances, but the type and common characteristics of these NKT stimulators remain poorly elucidated. Here we summarize three types of molecules that lead to NKT-cell activation (**Figure 1**), and briefly discuss their roles in the process of liver diseases.

### α-GalCer and analogues

Previous studies have shown that lipid antigens are presented to NKT cells through CD1d located on the surface of dendritic cells. Among all the lipid antigens, α-GalCer (also known as KRN7000), a synthetic component, is the first to be experimentally confirmed to potentiate NKT cells, both *in vitro* and *in vivo* (5, 13, 14).This glycolipid was discovered in an extract of the marine sponge Agelasmauritianus, and its effect on NKT in both *in vitro* and *in vivo* activation is widely reported. After α-GalCer administration, there are detectable increases in the number of NKT cells (15)and NKT-derived cytokines (14)(TNF (tumor necrosis factor), IFN-γ (interferon-γ), IL-12, etc.) as well as a degranulation marker (CD107a) (20), and symptoms of experimental animals improve or worsen accordingly (21, 22). Some researchers have also proposed an α-GalCer-based therapy for infection and autoimmune diseases (23, 24), but this proposal has been met with the opposing argument that administration of α-GalCer is also likely to induce hepatocyte damage (15)and NKT-cell anergy (25).

In many types of liver diseases, administration of exogenous α-GalCer changes their pathophysiological process. For example, α-GalCer-induced NKT activation is responsible for exacerbation of ALDs (26). The anti-tumor activity of α-GalCer in the liver is also demonstrated in mice experiments (27). Interestingly, in the mouse model of CCl_4_-induced acute liver injury, natural activation of NKT cells ameliorates liver damage and inflammation, possibly by suppressing HSC (hepatic stellate cell) activation, while α-GalCer-induced NKT activation accelerated acute liver injury, inflammation and fibrosis (28). Considering its dual effects on liver diseases and hepatic toxicity, further trial of α-GalCer is needed before clinical use.

Furthermore, analogues of α-GalCer are studied for their potential to activate NKT cells. In 2008, Jonathan D. Silk et al. (29)reported that threitolceramide- (ThrCer-) induced activation of NKT cells. In addition, researchers have managed to createα-GalCer analogues artificially, including α-GalCer-diol (with added hydroxyl groups in the acyl chain compared to α-GalCer, Juan Ma et al., 2020) (30)and6′-modified α-GalCer analogues (Matthias Trappeniers et al., 2008) (31). Hopefully, with careful design, these analogues will be applicable as preventative and therapeutic vaccine adjuvants (32, 33).

However, it is worth noting that not allα-GalCer analogues have the potential to activate NKT cells. For instance, in 2005, Jochen Mattner et al. (8)found that injection of α-glucuronosylceramide (PBS 30) or galacturonosylceramide (PBS 59) in mice led to the proliferation of NKT cells, but β-glucuronosylceramide (PBS 50) did not.

Other lipid antigens that activate NKT cells include glycosphingolipid isoglobotrihexosylceramide (iGb3), an endogenous antigen synthesized in the endoplasmic reticulum (ER) and Golgicomplex (34, 35), and bacteria-derived lipopolysaccharide (LPS), a ligand for Toll-like receptor 4 (TLR4) expressed on NKT cells, which corresponds with the roles that NKT cells play during exogenous infection (8, 36, 37).

### Interleukins

Interleukins are a group of cytokines that are partially secreted by NKT cells, and some of them have biological effects on NKT cells. In brief, interleukins can activate NKT cells include IL-2, 12, 15, 18, 21, 27, and 33 (**Table 1**).

**Table 1:** Reported interleukin-induced NKT cells activation.

Interleukins	Mechanism	Effects	References
**IL-2**	No data found	Increased number of NKT cells and enhanced secretion of IFN-γ	(38–40)
**IL-12**	No data found	Enhanced Th1 responses	(41)
**IL-15**	NF-κB signaling	Enhanced proliferation and homeostasis	(40, 42–45)
**IL-18**	NF-κB signaling	Enhanced Th1 and Th2 responses	(16–18)
**IL-21**	Autocrine	Enhanced Th2 responses	(46, 47)
**IL-27**	Modulate IL-12 secretion of DCs	Enhanced maintenance and recruitment of NKT cells	(48)
**IL-33**	IL-33/ST2L interaction	Enhanced secretion of IFN-γ and FasL expression	(49)

Interleukins activate NKT cells by binding to receptors on the cell surface, and many of the working mechanisms of functioning interleukins remain elusive. Activated NKT cells secrete large amounts of Th1/Th2 cytokines, which could be modulated by administration of the interleukins mentioned above, indicating that these interleukins have a profound impact on NKT-cell activation.

IL-2 is found effective for stimulating NKT cells both *in vitro* and *in vivo* in many studies focused on mice. Co-administration of IL-2, 12 and 18 results in a stronger ability of NKT cells to secrete IFN-γ (38). Small amounts of IL-2 cDNA (complementary DNA) increases the number of NKT cells *in vivo* (39), and potentiates the effect of α-GalCer (50). Also, studies found that IL-2 enhances the effect of NKT activation by α-GalCer, but administration of IL-12 alone is not enough to potentiate NKT cells (40). In addition, exogenous IL-2 and/or IL-15 partially overcome the hyporeponsiveness of iNKT cells in chronic HBV patients (51).

Murine models showed that IL-15, partly from Kupffer cells, facilitates the proliferation and maintain the homeostasis of NKT cells (42, 43). IL-15 can potentiate the α-GalCer-stimulated NKT expansion (40). Some researchers have found that IL-15-related NKT activation is associated with the NF-κB signaling pathway, but the exact mechanism remains controversial. Vallabhapurapu S et al. claimed that IL-15-related NKT activation is dependent on the NF-κB signaling pathway, because they found that RelA, a member of Rel/NF-κB family, controls IL-15 signaling by regulating IL-15Rα chain and γC chain, and deficiency of RelA blocks NKT activation by administration of IL-15 (44). However, Locatelli I et al. believed NF-κB deficiency might stimulate NKT recruitment by promoting IL-15 activity (45).Mice experiments showed that the IL-18/IL-18R (IL-18 receptor) axis functions *via* a rapid NF-κB signaling pathway, directly enhancing IL-4 production by NKT cells. Unlike IL-12,which mainly promotes Th1 response (41), IL-18 stimulates Th1 and Th2 responses simultaneously (16–18); hence the two kinds of cytokines are sometimes co-administered for their combined activation effects (52). Also, we would like to point out that continuous stimulation of IL-18 may result in impaired long-term NKT activation, which is important during clinical practice.

Intriguingly, we noticed an autocrine phenomenon with regard to IL-21, namely that not only does IL-21 enhance the survival of NKT cells, it is also secreted by NKT cells after CD3 and CD28 administration. NKT cells activated by IL-21 exhibit higher granzyme and IL-4 expression (46). Some researchers also report witnessing less IFN-γ and TNF production by NKT cells, indicating that IL-21 leads to “Th1 to Th2” cytokine transformation (47), though this conclusion needs more support. Moreover, α-Galcerhas been found to coordinate with interleukins, including IL-18 and IL-21 (17, 46).

IL-27 and IL-33 also contribute to activation of NKT cells. IL-27 modulates IL-12 secretion of dendritic cells, thus indirectly enhancing maintenance and recruitment of NKT cells (48). IL-33 binds with ST2L (the suppressor of tumorgenicity 2 ligand, and also the receptor of IL-33) on NKT cells to promote IFN-γ secretion as well as FasL expression (49).

Some researchers have studied the functions of interleukins on liver NKT cells and examined them as possible treatment methods. For example, administration of IL-18 potentiates the cytotoxicity of hepatic NKT cells in a perforin-dependent way (53). Co-administration of IL-12 and IL-18 triggers higher IFN-γ release from NKT cells than either administered alone, which demonstrates a higher efficiency for killing liver tumors (54). However, extra work is urgently needed to investigate the effects of interleukins on hepatic NKT cells and their clinical values.

### Chemokines

The chemokine superfamily was first discovered in the late 1980s to play a role in inflammation. The protein superfamily consists of four groups, namely XC, CC, CXC, and CX3C, a categorization based on the discrete location of cysteine residues on the initial sequence of the molecules. Chemokine receptors are defined as a group of seven transmembrane-spanning G-protein-coupled receptors (GPCRs), having no one-on-one match with their ligands (55–57). Chemokines are tightly associated with the maturation and localization of NKT cells. The effective ligands and receptors are summarized in **Table 2**.

**Table 2:** Different chemokines on NKT cell activation/inactivation.

Chemokine subgroups	Ligands/Receptors	Functions	References
**XC**	No data found	No data found	/
**CC**	CCL2/CCR2	Suppress NKT cells (by recruiting NKT cells to spleen and preventing IL-4 secretion)	(58–60)
	CCL3/unknown	Recruit NKT cells	(61)
	CCL4/CCR5	Recruit NKT cells; activation-induced apoptosis	(61, 62)
	Unknown/CCR1	Recruit NKT cells	(60)
**CXC**	CXCL16/CXCR6	Recruit NKT cells; promote IFN-γ and IL-4 secretion of NKT cells	(63–65)
	CXCL13/CXCR5	Suppress NKT cells	(66)
**CX3C**	CX3C1/CX3CR1	Enhance NKT cells trafficking; define NKT sublineages	(67)

Of the four chemokine subgroups listed in **Table 2**, XC and CX3C have seldom been studied since their discovery; thus, there are very few articles concerning their functions on NKT cells. XC, interestingly, has not been reported so far to potentiate NKT cells. In contrast, CX3C1/CX3CR1 is considered to take part in NKT-cell trafficking within the thymus, but this function may not be of vital importance as CX3CR1-deficient mice do not show NKT-cell developmental disability (67). Also, some researchers hold opposing views on the NKT-cell-activating function of CX3C1/CX3CR1 (68). However, CX3CR1 expression on the cell surface can be utilized to define NKT subtype present in the thymus and peripheral organs (67).

Unlike XC and CX3C, the functions of the other two subgroups are known in more details. In mice models, CCL2, also known as MCP-1, recruits NKT cells to peripheral organs such as the spleen (58) and exerts an anti-inflammatory effect by interacting with CCR2 to prevent IL-4 secretion of NKT cells, which demonstrates a hepatoprotective effect in the liver (59). CCL3 and CCL4, secreted by activated dendritic cells, also attract NKT cells. This effect is accompanied by CXCR3 ligands (CXCL9-11) which derive from the same dendritic cells (61). CCL4 has also been found to induce distinct chemotaxis in different NKT subgroups, attracting CCR5-expressing cells in particular (69). Lack of CCR5 in mice promotes fulminant liver failure because of exacerbated inflammatory responses related to a higher amount of IL-4 from NKT cells that fail to go through apoptosis after activation (62), suggesting a role for CCR5 in NKT-cell regulation. Another CC chemokine receptor on NKT cells surface is CCR1, which together with CCR5 recognizes ligands that come from activated macrophages and dendritic cells (60).

The last type, CXC, is the most comprehensively studied at present, especially CXCR6 and its ligand CXCL16. In short, CXCR6/CXCL16 functions on the distribution rather than maturation of NKT cells. Animal research shows that CXCR6 expression of NKT cells is elevated upon NKT-cell activation, but is not indispensable for NKT-cell development within the thymus, as CXCR6-challenged mice do not show reduced numbers of thymic NKT cells. However, CXCR6 is closely related to localization of NKT cells because of its interaction with CXCL16, which resides on target organs such as the spleen and liver (70). As a result, CXCR6-deficient mice possess fewer NKT cells in their livers, making them more susceptible to infection (63, 70). The CXCR6/CXCL16 reaction boosts IFN-γ and IL-4 release from NKT cells, enhancing inflammatory response (64, 65). In particular, CXCR6/CXCL16 is involved in many liver diseases. For example, CXCR6/CXCL16 expressions greatly increase during liver inflammation (71). Hepatocytes produce CXCL16 in non-alcoholic fatty liver disease (NAFLDs), which ameliorates inflammation and fibrosis (72, 73). In contrast, CXCR5, along with its ligand CXCL13, is reported to reduce NKT-cell activation (66).

## The relationship between NKT cells and other cells in liver disease

In the liver, NKT cells have close connections with other cells including hepatocytes (normal liver cells), dendritic cells, macrophages (Kupffer cells), T cells, and B cells, and are able to regulate their functions during innate and acquired immune reactions. This connection is achieved through either direct contact or secretion of cytokines. Evidence shows the significance of this connection because changes in how NKT cells interact with other cells can be found in liver disease and may lead to severe dysfunction of the organ. Here we summarize the ways in which NKT cells coordinate with other liver-resident cells.

### NKT cells and hepatocytes

As mentioned above, NKT cells are activated by lipid antigens through CD1d molecules which, in the liver, are expressed on macrophages, dendritic cells, and hepatocytes. CD1d then presents the antigens to the TCR on NKT cells. A decrease in CD1d on hepatocytes results in dysfunction of NKT cells (74). Some studies based on HBV transgenic mice find that during liver diseases such as HBV infection, CD1d expression is elevated on injured hepatocytes, rather than macrophages (75). Meanwhile, hepatocyte-derived IL-7 is also important in the maintenance of NKT cells, which indicates that hepatocytes play a role in the development and maintenance of the immune system (76).

**Figure 2:**
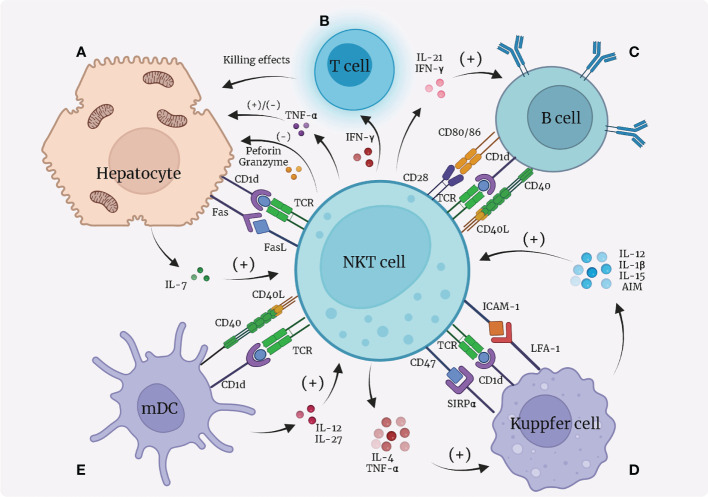
Interactions between NKT cells and hepatocytes, dendritic cells, Kupffer cells, and B cells. **(A)** Hepatocytes present lipid antigens to TCR on NKT cells *via* the CD1d-dependent pathway; hepatocytes release IL-7 to activate NKT cells; NKT cells secrete TNF-α that has dual functions on hepatocytes; NKT cells express FasL and induce Fas expression on hepatocytes, leading to apoptosis of hepatocytes. **(B)** NKT cells secrete IFN-γ to recruit T cells to kill hepatocytes. **(C)** B cells present lipid antigens to NKT cells through CD1d; NKT cells and B cells contact each other directly through CD40L/CD40 and CD28/CD80, 86; NKT cells secrete IL-21 and IFN-γ to promote B cells. **(D)** Kupffer cells present lipid antigens to TCR on NKT cells *via* the CD1d-dependent pathway; Kupffer cells express LFA-1 and SIRPα, which bind with ICAM-1 and CD47, respectively, on NKT cells to activate NKT cells; Kupffer cells secrete IL-12, IL-1β, IL-15, and AIM to recruit and promote NKT cells; NKT cells in return produce pro-inflammatory IL-4 and IFN-γ to function on Kupffer cells. Notably, over-stimulation of NKT cells by Kupffer cells leads to apoptosis of NKT cells. **(E) **Dendritic cells (especially myeloid dendritic cells, mDC) present lipid antigens *via* CD1d towards NKT cells to activate NKT cells; dendritic cells secrete IL-27 and IL-12 to activate NKT cells; NKT cells express CD40L to bind with CD40L and reciprocally benefit dendritic cells. mDC, myeloid dendritic cells; IL, interleukin; TNF, tumor necrosis factor; IFN, interferon; SIRP, signal regulatory protein; LFA, lymphocyte function-associated antigen; ICAM, intercellular adhesion molecule; AIM, apoptosis inhibitor expressed by macrophages.

NKT cells attack hepatocytes by expressing FasL, perforin, and granzymes, but their main effects on hepatocytes are achieved by producing Th1 cytokines, especially TNF-α and IFN-γ. Upon activation, NKT cells start to release more TNF-α that directly interacts with TNF receptor 1 (TNFR1)expressed on hepatocytes, on which this molecule has a dual effect, either promoting hepatocyte death or regeneration indifferent contexts (77). Increased level of NKT-derived TNF-α is responsible for exacerbation of α-GalCer-induced liver damage (26). However, in mice that underwent partial hepatectomy, TNF-α promotes regeneration of hepatocytes (78). The interactions between NKT cells and hepatocytes are also tightly associated with a wide range of liver diseases. For instance, in autoimmune liver diseases, NKT cells release death signals to hepatocytes through FasL pathway, and secrete TNF-α, perforin and granzymes in synchronization, promoting the process of autoimmune liver diseases (79). In ALDs, NKT cells also play the role of killing hepatocytes (80). In 2014, Monika Julia Wolf et al. (81)found that TNFSF14 (TNF superfamily 14, also referred to as LIGHT) secreted by NKT cells is responsible for enhanced lipid uptake of hepatocytes as well as liver damage, causing an enhanced possibility of NAFLDs in mice (**Figure 2A**).

IFN-γ expression is also increased in activated NKT cells. During HCV infection, IFN-γ induces liver sinusoidal endothelial cells to produce CXCL9 and CXCL10 that bind to and recruit CXCR-positive T cells. As a result, more T cells start to locate in the infected liver and negatively affect hepatocytes (82). Moreover, IFN-γ stimulates hepatocytes to express a higher number of Fas, causing liver cell apoptosis after binding with FasL on NKT cells (**Figure 2B**) (83).

### NKT cells and B cells

The interactions between NKT cells and B cells mainly lead to strengthened capacity of B cells. Animal research suggested that CD1d loaded with lipid antigens from B cells surface is a source for NKT-cell activation (84, 85). In return, NKT cells offer helper signals for B cells by expression of CD40L and CD28, which bind to CD40 and CD80/86, respectively, on B cells (86). Secretion of IL-21 (87) and IFN-γ (86) also play a role in B-cell activation. In addition, NKT cells can indirectly enhance B cells by communicating with dendritic and CD4+ T cells (84, 85, 88). Interestingly, stimulation of NKT cells also leads to recruitment of regulatory B cells to the liver that suppress inflammation (**Figure 2C**) (89). However, up to now, it remains poorly studied how the interactions between NKT and B cells contribute to the pathogenesis of hepatic diseases.

### NKT cells and macrophages (Kupffer cells)

The interaction between NKT cells and macrophages is relatively complex as it involves multiple surface and secreted molecules. As one of the main APCs in the liver, macrophages (Kupffer cells) connect with NKT cells in a CD1d-restricted manner. The CD1d molecule located on the surface of Kupffer cells presents exogenous lipid antigens to TCR on NKT cells, leading to NKT-cell activation (90).

Another pivotal means of Kupffer/NKT interaction is through the LFA-1/ICAM-1 pathway. LFA-1 (lymphocyte function-associated antigen 1) and ICAM-1 (intercellular adhesion molecule 1) are resident on the surface of Kupffer cells and NKT cells, respectively, and bind to each other with high affinity. Aside from NKT-cell activation, Kupffer cells are also reported to show quicker iNOS (inducible nitric oxide synthase) and NO synthesis, indicating mutual activating functions between NKT cells and Kupffer cells (91). Notably, over-stimulation of NKT cells by Kupffer cells can result in activation-induced apoptosis and necrosis of NKT cells (90). Signal regulatory protein α (SIRPα) on Kupffer cells binding to CD47 on NKT cells also enhances the function of NKT cells (92).

Kupffer cells secrete many types of cytokines that have biological functions on NKT cells, mostly different interleukins. Kupffer-cell-derived IL-12, IL-1β, and IL-15 are thought to recruit NKT cells, promote NKT-cell activation, and participate in the maintenance of NKT cells (42, 93–95). AIM (“apoptosis inhibitor expressed by macrophages”, also referred to as CD5L), a protein that is normally considered to inhibit apoptosis of CD4+CD8+ double-positive thymocytes, is secreted by Kupffer cells to protect NKT cells from apoptosis (96). On the other hand, NKT cells are capable of producing large quantities of pro-inflammatory IL-4 and IFN-γ, which are associated with granuloma formation around infected Kupffer cells (97).

In conclusion, we believe the relationship between NKT cells and Kupffer cells is reciprocal, enhancing both NKT and Kupffer cells (**Figure 2D**). In hepatic diseases like inflammation (93, 98), alcoholic liver injury (94) and infection (92), the interactions between Kupffer cells and NKT cells play an indispensable part.

### NKT cells and dendritic cells

As a type of APC, dendritic cells participate in lipid antigen (such as α-Galcer) presentation *via* CD1d towards NKT cells, which leads to NKT-cell activation (99, 100), just like hepatic macrophages do. Dendritic cells also secrete cytokines including IL-27 (48) and IL-12 (101) that both have a positive impact on NKT cells. Notably, only myeloid dendritic cells (mDC), not all dendritic cells, mediate activation of NKT cells, whereas plasmacytoid dendritic cells (pDC) are likely to cause tolerance of NKT cells in a way concerning the activation of type II NKT cells (6). In addition, NKT cells also function as promoters of dendritic cells by expressing CD40L that binds with CD40 on the surface of dendritic cells, forming a reciprocal activating loop (5, 101). It has been observed that dendritic cells respond to TLR stimulation more actively in the presence of NKT cells (**Figure 2E**) (102, 103).

The positive influence of dendritic cells on NKT cells might provide an insight into treatment of liver diseases. In 2007, Tomohide Tatsumi et al. (104)demonstrated with mice models that α-GalCer-pulsed dendritic cells suppressed liver tumor by activating NK cells, and they proposed that NKT cells might also take a part. Hopefully, future research might provide a more explicit answer.

### NKT cells and T cells

T cells consist of a wide range of different cell subgroups including CD8+ T cells, CD4+ T cells, and regulatory T cells (Tregs), each having distinct immune bioactivity.

The interaction between NKT cells and CD8+ T cells seems confusing as researchers have obtained contradictory experimental results. Some people believe NKT cells boost CD8+ T cells just like they do CD4+ T cells, *via* CD40/CD40L signaling and secretion of cytokines such as IL-4 and IL-13. Activated CD8+ T cells then secrete IFN-γ, a pro-inflammatory cytokine (105, 106). However, other researchers have discovered an inhibitory effect of NKT cells on CD8+ T cells in animal experiments. IFN-γ secreted by activated CD8+ T cells allows NKT cells to produce IL-4 and IL-13, which in turn inhibit CD8+ T cell activity by harassing their chemotaxis to CCL5. Also, NKT cells indirectly suppress CD8+ T cells by potentiating regulatory T cells (Tregs) (107).

The effects of NKT cells on CD4+ T cells are mainly positive, leading to enhanced IFN-γ secretion (105, 108). Normally, CD4+ T cells are the main producers of IL-10, a cytokine that has anti-inflammatory effects, but their IL-10 secretion is profoundly inhibited after activation from NKT cells (105). This indicates that activation of CD4+ T cells by NKT cells may be pro-inflammatory. Dendritic cells are also said to participate in NKT/T crosstalk, enhancing both CD4+ and CD8+ T cells (106). Moreover, NKT-cell-induced Treg activation plays an important role in the depletion of CD4+ T cells (109).

**Figure 3:**
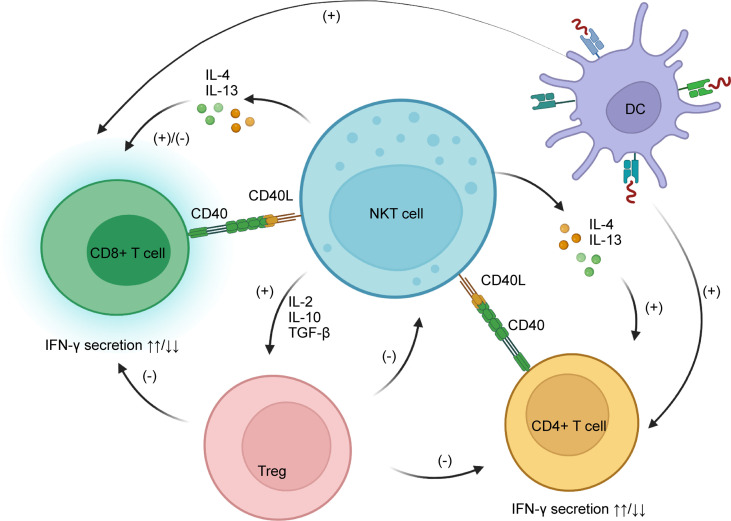
NKT cells interact with CD8+ and CD4+ T cells through CD40/CD40L and secretion of IL-4 and IL-13. The main functions of NKT cells on CD4+ T cells are stimulatory, while on CD8+ T cells they are both stimulatory and inhibitory, as NKT cells can harass their chemotaxis. NKT cells potentiate Tregs by secreting IL-2, IL-10, and TGF-β; Tregs have a negative impact on CD8+ and CD4+ T cells as well as NKT cells. IL, interleukin; TGF, tumor growth factor; Treg, regulatory T cells.

Regulatory T cells (Tregs), on the other hand, are a special subgroup of T cells that can suppress immune reactions. Some studies show a negative feedback relationship between NKT cells and Tregs. Activated NKT cells stimulate activation of Tregs by secreting higher amounts of cytokines such as IL-2, IL-10, and TGF-β. Tregs then suppress the proliferation and functions of NKT cells, reducing their cytotoxic activity. Interestingly, the inhibitory effects of Tregs on NKT cells are achieved in a CD1d-dependent manner that requires direct cell-to-cell contact (110, 111), rather than TGF-β and IL-10, as the suppression of NKT cells continues even after neutralization of the two cytokines (109, 111). This feedback mechanism is likely to prevent over-stimulation of NKT cells and the disastrous cascade immune reactions that could ensue (see **Table 3** and **Figure 3**).

**Table 3:** Interactions between NKT cells and different subgroups of T cells.

T cell subsets	Interaction with NKT cells	Results on T cells	Results on NKT cells	References
**CD8+ T cells**	CD40/CD40L, IL-4, IL-13	Enhanced IFN-γ secretion, inhibited chemotaxis to CCL5	Enhanced IL-4 and IL-13 secretion	(105–107)
**CD4+ T cells**	CD40/CD40L, IL-4, IL-13	Enhanced IFN-γ secretion, reduced IL-10 secretion	Decreased suppression of IL-10 on NKT cells	(105, 106, 108)
**Regulatory T cells (Treg)**	Cell-to-cell contact	Enhanced secretion of IL-2, IL-10 and TGF-β (which functions negatively on CD8+ and CD4+ T cells)	Impaired proliferation	(110, 111)

The interactions between NKT and T cells play a pivotal role in liver diseases. For example, during HBV infection in a transgenic mouse model, NKT cells promotes the proliferation of HBV-specific CD8+ T cells (112), and blockade of NKG2D expression prevents hepatitis induced by T cells (113). These results respond with the investigation which found that HCV-specific T cell response comes in accordance of NKG2D expression on NKT cells in healthcare workers who were exposed to small amounts of HCV but showed no obvious liver damage (114). In autoimmune liver disease and NAFLDs, NKT cells contribute to the recruitment of T cells, and potentiate their biological functions (79, 115, 116). As a result, NKT cells are considered as a factor for exacerbation of these two diseases. However, in autoimmune liver diseases, NKT cells promote the activation of Tregs, which ameliorates the killing effect of T cells (117).

### NKT cells and other cells

Besides hepatocytes, dendritic cells, macrophages (Kupffer cells), T cells, and B cells, hepatic NKT cells also have close relationship with neutrophils, hepatic stellate cells, and NK cells. These interactions have a significant influence in modulating inflammation as well as immune tolerance, and play a role in the processes of liver cirrhosis and cancerous proliferation. We outline these cell interactions as follows.

NKT cells secrete IL-4 to recruit and promote accumulation of neutrophils, thus enhancing hepatitis and liver fibrosis (115, 118). Additionally, NKT-cell-derived IFN-γ acts as a potent suppressor of neutrophils by inducing apoptosis (118). This may be to prevent over-activation of pro-inflammatory responses.

HSC activation is among the causes of liver fibrosis as it enhances synthesis and accumulation of collagen and extracellular matrix (119). NKT cells can either have a stimulatory or inhibitory effect on HSCs. Normally, NKT cells enhance the growth of HSCs *via* Hh (Hedgehog) signaling pathway and secreting OPN (osteopontin) (81, 120, 121), but under certain circumstances NKT cells induce HSCs apoptosis *via* FasL (122). (See 4.3”alcoholic liver diseases (ALDs)” and 4.4”non-alcoholic fatty liver diseases (NAFLDs)”).

Generally, NKT cells are considered to enhance the activation of NK cells. Tomonori Iyoda et al. (123)found activated NKT cells induce NKG2D and DNAM-1 (also known as CD226) expression on NK cells that are necessary for the anti-tumor effects of NK cells. NKT-cell activation also leads to improved cytokine production (e.g IFN-γ) and killing activity of NK cells through the mTOR (mechanistic target of rapamycin) pathway, which brings about enhanced anti-pathogen capacity (124, 125). However, some researchers have also reported an inhibitory effect of NKT cells on IFN-γ secretion of NK cells after alcohol intake (126).

## NKT cells in liver diseases

Given all the functions of NKT cells on other cells within the liver, it is easily deduced that NKT cells make an enormous contribution to the pathogenesis and progression of many kinds of liver disease ranging from autoimmune hepatitis to hepatoma. With more insights into how NKT cells work in these diseases, hopefully new methods to treat or cure liver diseases will be discovered. For example, tazarotene, a RAR-γ (retinoic acid receptor-γ) agonist that inhibits NKT-cell proliferation, as well as cytokine release, is tested in mice for treatment of liver steatosis and fibrosis (127). Below, we discuss how NKT cells play their role in autoimmune liver diseases, alcoholic liver diseases (ALDs), non-alcoholic fatty liver diseases (NAFLDs), and liver tumors.

### HBV and HCV infection

HBV and HCV infections are two important reasons for liver damage all across the world, affecting approximately 250 million (128) and 80 million (129) people respectively. Thus, research aimed at fighting against viral hepatitis has become a global task. In this chapter, we will review the roles that NKT cells play during the pathophysiological process of HBV and HCV infection.

During HBV infection, hepatocytes that are invaded by virus would present lipid antigens, namely lysophosphatidylethanolamine, to NKT cells *via* CD1d (130). This leads to NKT activation, causing an elevated amount of IFN-γ, which mediates anti-viral effects (131) but also results in liver damage (132) in mice models. IFN-γ inhibits the proliferation of hepatocytes by inducing apoptosis and negatively regulating cell cycle (75). In addition, NKT cells inhibit HBV by promoting the activation of cytotoxic T lymphocytes (CTL) (112). Blockade of NKG2D is found to ameliorate acute HBV hepatitis both *in vitro* and *in vivo (* 113). In a retrospective investigation in 2009, India, researchers found that the amount of NKT cells is smaller in fulminant HBV liver failure than acute HBV patients, indicating the role that NKT cells play in limiting HBV infection (133). Notably, any factors that hinder the presentation of lipid antigens of hepatocytes to NKT cells, such as deficiency of NKT cells or CD1d or dysfunction of ER-associated lipid transfer, would result in a delayed anti-viral reaction (130).

However, data collected from clinical patients demonstrated that there is a decreased density (134) and down-regulated function (135) of NKT cells for chronic HBV infection compared to acute HBV infection. Both animal and human studies suggested that NKT cells are associated with over-activation of HSC and excessive healing during HBV infection, which increases the possibility of liver cirrhosis (136, 137). Moreover, the number of NKT cells is positively correlated with the quantity of HBV during chronic HBV infection, and a decrease in NKT number is witnessed after effective anti-viral treatment. This indicates NKT density as a potential marker for evaluating anti-HBV treatments (138).

To this day, plenty of novel NKT-related treating methods of HBV have been experimented on cells or clinical trials. For example, α-GalCer was found to inhibit HBV replication by directly activating NKT cells in mice (131), but clinical trials showed pessimistic results, as administration of α-GalCer alone even decreases NKT density and does not influence density of HBV DNA (139). Also, the function of α-GalCer on NKT activation decreases during chronic HBV infection, but this phenomenon is partly reversible after administration of exogenous IL-2 and/or IL-15 (51). Other ways to activate hepatic NKT cells and inhibit HBV replication include IL-18 (140), thymosin-α1 (141), β-glycosphingolipids (142), CD28/CD80 (143) activation and PD-1/PD-L1 blockade (143), but these methods have not received clinical confirmation yet.

NKT cells also participate in the process of HCV infection. Elevated expression of CD1d on infected biliary cells promotes the activation of NKT cells (144), leading to secretion of cytokines including IL-4 which recruits T cells and perforin and granzyme which mediate liver damage (145). Notably, some researchers claimed that the number of NKT cells in peripheral blood decreases in patients infected with HCV (146, 147), but more researchers did not find significant changes in number of NKT cells (148–150), although hepatic NKT cells showed enhanced activity (147), producing a higher amount of IL-13 that has a pro-Th2 effect (150). The functions of NKT cells during HCV are associated with macrophages and T cells. Macrophages (Kupffer cells) in the liver secrete significantly more IL-15 that boost NKT activation (95). In healthcare workers who were continuously exposed to small amounts of HCV but did not develop symptoms, NKT cells were found to be activated in a way related to specific T cells activation, indicating the protective effect of NKT cells against HCV is partly associated with T cells (114). In patients with chronic HCV, the sustained response to IFN plus ribavirin therapy is associated with elevated dynamism of NK and NKT cells, suggesting NKT cells play a vital role in anti-HCV reaction (151). In addition, in pregnant women infected by HCV, density of NKT cells increase in placenta tissues, which is thought to be responsible for preterm birth (152).

Preclinical studies proposed novel NKT-related anti-HCV therapy including the administration of IFN-α (153) and IL-2/OKT3 (a CD3-specific mAb) (154), which leads to NKT activation and up-regulated IFN-γ expression that inhibit virus replication in mice. Moreover, a clinical trial experimented oral administration of hepatocyte-extracted proteins and HBV or HCV proteins to figure out their anti-viral functions in chronic HBV or HCV patients. Results showed that all patients experienced increased number of NKT cells for at least 2-fold, and histological necro inflammatory score improved in 4/13 (30.7%) and 2/12 (17%) patients of HBV and HCV, respectively (155).

### Autoimmune liver diseases

There are three main types of autoimmune liver disease, namely autoimmune hepatitis (AIH), primary biliary cirrhosis (PBC), and primary sclerosing cholangitis (PSC), which are associated with destruction of hepatic parenchyma, small intrahepatic bile ducts, and large bile ducts, respectively. Liver NKT cells primarily reside in liver sinusoids. Numerous studies have found that NKT cells are closely associated with all three types of autoimmune disease (79, 115, 117, 156).

Interestingly, liver NKT cells can either promote or combat autoimmune liver diseases depending on their down-stream target cells (**Table 4**). Besides, NKT cells promote the development of hepatocyte injury in three ways (1): NKT cells directly kill hepatocytes by expressing FasL and secrete TNF-α, perforin and granzyme B; (2) NKT-derived IFN-γ induces Th0-cell transduction into Th1 cells and CD8+ T-cell transduction into CTLs that bind to the MHC I molecule on the surface of hepatocytes; (3) NKT cells secrete IL-4 that turns Th0 cells into Th2 cells, enhancing B-cell-producing autoimmune antibodies(79). Furthermore, TNF-α and IFN-γ are involved in recruitment of functional T cells, and IL-4 probably promotes neutrophil infiltration within the liver (115). NKT cells are also potent activators of Tregs, which have a negative effect on immune response, thus mitigating autoimmune liver injury (160). Statistics show that simultaneous suppression of NKT cells and promotion of Tregs is helpful for mitigating autoimmune liver injuries in experimental animal models (161).

**Table 4:** Functions of NKT cells in autoimmune liver diseases, alcoholic liver diseases (ALDs) and non-alcoholic fatty liver diseases (NAFLDs).

Disease type	Role of NKT	Mechanisms	References
**HBV**	Inhibitors of HBV replication	Inhibit hepatocyte proliferation (by secreting IFN-γ that induces apoptosis and negatively modulates cell cycle)	(131, 132)
		Promote the activation of CTL	(112)
	Destructive factors	Cause liver damage	(132)
		Cause over-activation of HSCs and excessive healing, promoting cirrhosis	(136, 137)
**HCV**	Inhibitors of HCV replication	Death of infected liver cells (by perforin and granzyme)	(145)
		Recruit and activate T cells	(145)
**Autoimmune liver diseases**	Promoters of autoimmune liver diseases	Kill hepatocytes (via FasL, TNF-α, perforin, granzyme B)	(79)
		IFN-γ (induce Th0→Th1 and CD8+ T cells →CTL transformation, recruit T cells)	
		IL-4 (induce Th0→Th2 transformation that promotes B cells to produce antibodies, recruit neutrophils)	
		TNF-α (recruit T cells)	(115)
	Inhibitors of autoimmune liver diseases	Activate Tregs	(117)
**Alcoholic liver diseases (ALDs)**	Promoters of ALDs	NKT cells recruit neutrophils (via TNF-α, etc.)	(94, 157, 158)
		NKT cells mediate death of hepatocytes (via FasL)	(80)
		NKT cells inhibit IFN-γ secretion of NK cells	(126)
	Inhibitors of ALDs	NKT cells suppress HSCs (via FasL, IFN-γ) in early stage of ALDs	(122)
**Non-alcoholic fatty liver diseases (NAFLDs)**	Promoters of NAFLDs	NKT cells improve insulin resistance	(81)
	Inhibitors of NAFLDs	NKT cells enhance lipid intake of hepatocytes (via secretion of LIGHT)	(81, 120, 121)
		NKT cells activate HSCs (via OPN, Hh pathway)	(116)
		NKT cells recruit CD8+ T cells and macrophages	(120, 159)

Apart from inflammation, NKT cells are likely to play a role in liver fibrosis resulting from autoimmune liver diseases. IL-4 and IL-13 from activated NKT cells promote liver fibrosis, suggesting a role for NKT cells in cirrhosis resulting from chronic autoimmune liver injury (77). However, this fibrinogenic effect requires further examination (115).

Given the roles that NKT cells play during autoimmune liver diseases, researchers have been experimenting on modulating NKT cells to find treatments for the disease. In recent years, several substances have been found effective in mice for alleviating concanavalin A (Con A)-induced autoimmune hepatitis partly by inhibiting NKT cells and related production of inflammatory cytokines, including mitochondrial-targeted ubiquinone(MitoQ) (162), diammonium glycyrrhizinate (161) and secoemestrin C (163). Gene modulation such as C6orf120 knockout (164) is also used as a therapeutic target. However, these experiments were only done on mice, and lack of clinical statistics limits extensive application.

Other studies also suggested the role that type II NKT cells plays in autoimmune liver diseases. The number of type II NKT cells was up-regulated in both peripheral blood and liver during autoimmune liver diseases (165). Increased CD1d expression on T cells during autoimmune liver diseases results in activation of type II NKT cells and favors Th1 cytokine production over Th2 within type II NKT cells (166). It is important to understand the physiology of type II NKT cells as they might influence iNKT cells. For example, Ramesh C. Halder et al. reported that activation of type II NKT cells and pDCs are associated with recruitment of anergic iNKT cells (167).

### Alcoholic liver diseases

It is widely known that excessive consumption of alcohol ranks high among the risk factors for liver pathogenesis. Alcohol-induced liver diseases include alcoholic hepatitis, steatosis, cirrhosis, and hepatocellular carcinoma (HCC).

ALDs are closely linked to enhanced immune activation (168). Activated inflammatory responses are noticed quickly after alcohol intake with a higher NKT-cell concentration within the liver as well as mesenteric lymph nodes (169–171). Conversely, the absolute number of NKT cells in the whole body is decreased (172), which may be the result of NKT-cell recruitment to the liver and vast consumption of these cells.

Generally, NKT cells are thought to contribute to the development of ALDs, in contrast to type II NKT cells that are considered to attenuate ALDs (157). Binge-feeding with ethanol results in accumulation and activation of NKT cells combined with a higher expression of inflammatory and fibrotic genes in wild-type mice compared to their NKT-deficient counterparts (158). Evidence shows that alcohol potentiates α-Galcer stimulation of NKT cells by facilitating CD1d loading (171). IL-1β from Kupffer cells is also required for hepatic NKT-cell accumulation during ALDs (94).

Activated NKT cells recruit neutrophils by secreting TNF-α and up-regulating expression of neutrophil-attracting MCP-1 (monocyte chemoattractant protein-1, also known as CCL2) (94, 157), ICAM-1 (intercellular adhesion molecule-1), E-selectin, MIP-1α (macrophage inflammatory protein-1α, also known as CCL3), MIP-2, and osteopontin (OPN) (158). Also, NKT cells mediate apoptosis of hepatocytes by expression of FasL (80). Some researchers consider NK cells to be protective against ALDs by secretion of IFN-γ, while NKT cells inhibit this process. However, a subgroup of IL-10-secreting NKT cells (thus called NKT10) facilitates the protective effect of NK cells in ALDs (126).

Controversially, in the early stages of ALDs, NKT cells are likely to play an anti-fibrotic role by suppressing HSCs. The negative influence of NKT cells on HSCs is achieved through direct killing *via* the Fas/FasL pathway and IFN-γ production (122). Taken together, we conclude that with a few exceptions, NKT cells are mainly promoters of ALDs (**Table 4**).

Up to now, we have not found any results of NKT-based clinical trial for ALD therapy, but there are experiments done on mice, showing several promising molecules with therapeutic potential, including retinoids and sulfatide (157). These two substances alleviate ALDs by inhibition of NKT cells. Additionally, researchers found that prednisolone, a drug widely used to antagonize inflammation, exacerbates ALDs by inhibiting phagocytosis mediated by macrophages and neutrophils and hepatic regeneration, which provide an insight into management of steroid therapy (173).

### Non-alcoholic fatty liver diseases

As the name suggests, the most significant feature of non-alcoholic fatty liver diseases (NAFLDs) is abnormal lipid storage in the liver. NAFLDs include simple stenosis, non-alcoholic steatohepatitis (NASH), cirrhosis, and even liver cancer. In many studies that focus on NAFLDs, a high-fat feeding model is used. Statistics have demonstrated that a high-fat diet, especially one with high concentrations of saturated fatty acids and monounsaturated fatty acids rather than polyunsaturated fatty acids, is associated with liver inflammation, insulin resistance, and NAFLDs (64, 174, 175).

During NAFLDs, the number of NKT cells within the liver decreases. This is because: (1) endothelium stress leads to fewer CD1d’s and impaired lipid presentation (74); (2) Kupffer cells mediate apoptosis and necrosis of over-activated NKT cells and secrete IL-12 to suppress NKT cells (64, 90); (3) normally Kupffer cells-derived IL-15 is stimulating for NKT cells, but in NAFLDs IL-15 secretion is down-regulated (45, 176) and (4) norepinephrine (NE) concentration decreases (176). However, some studies also report an increase in the number of NKT cells in the late stages of NAFLDs, probably because of enhanced activating functions of Kupffer cells *via* the CD1d-dependent pathway, which is inconsistent with many other study results (120).

Notably, the effects of NKT cells on the development of NAFLDs are rather controversial. While NKT cells ameliorate NAFLDs, probably by improving insulin resistance (120, 159), they are also likely to play a pro-inflammatory role in NAFLDs. Some studies show that NKT cells secrete LIGHT (TNFSF14), which significantly enhances lipid intake of hepatocytes (81). Also, NKT cells lead to activation of HSCs in two ways: (1) they facilitate OPN (osteopontin) secretion; and (2) they promote the Hedgehog (Hh) signaling pathway (note that NKT cells are both inducers and targets of the Hh signaling pathway). HSC activation is associated with exacerbation of liver fibrosis or cirrhosis (81, 120, 121). Activated NKT cells recruit CD8+ T cells and macrophages, too (116). Overall, NKT may have a protective effect in the early stages of NAFLDs but a destructive effect in later stages (**Table 4**).

In 2017, a published clinical trial said oral administration of β-glucosylceramide improved the hepatic fat content by 14% in NASH patients, which is associated with a decrease in CD4+ and NKT cells, suggesting NKT cells as a possible therapeutic target (177). Mice models also indicate that oral administration of liver-extrated proteins (178), immunoglobulin G-enhanced colostrums (179) and PRX-106 (180) (a recombinant anti-TNF-αfusion protein). The number of hepatic NKT cells was increased in all these mice models.

### Liver tumors

Liver tumors are a global health problem that deprives millions of people of their lives. primary hepatic carcinoma (HCC) is the main type of liver tumor. Many studies have shown the tumor-suppressing effect of hepatic NKT cells. Understanding of the roles that NKT cells play in the pathogenesis of both primary and metastatic liver cancer is helpful for finding effective ways of treatment.

NKT cells participate in anti-tumor immune responses mainly by producing IFN-γ. Mice studies found that not only can activated NKT cells secrete IFN-γ, they also stimulate IFN-γ production from NK cells (12, 181). IFN-γ then functions on hepatic T cells and Kupffer cells, enhancing the cytotoxicity and phagocytosis of T cells and Kupffer cells, respectively (182, 183). NKT cells also participate in chemotaxis of T cells as they secrete T cell-recruiting chemokines such as MIP-1α, MIP-1, and IL-8. IFN-γ up-regulates CXCR3 expression of T cells, potentiating T-cell recruitment. Moreover, NKT cells directly mediate the death of tumor cells through FasL, perforin, and granzyme (183). On the other hand, some researchers do not perceive NKT cells as necessary in anti-tumor immunity (184).

Activation of NKT cells in the background of hepatic cancer is closely associated with dendritic cells and Kupffer cells. Dendritic cells communicate with NKT cells in a CD40/CD40L-dependent way. Up-regulated expression of CD40L in NKT cells potentiates DC cells, leading to secretion of IL-12 that in turn activates NKT cells (101). Kupffer cells are also sources of IL-12 (182). Importantly, IL-12-induced NKT activation is linked to reduced primary hepatic tumor and less metastasis to the liver (185) As a result, IL-12-based therapy has been proposed and examined by many researchers (186, 187).

Interestingly, activation of NKT cells is also dependent on CXCR6/CXCL16 interaction, as deficiency of CXCR6 or neutralization of CXCL16 cause hepatic cancer to deteriorate. CXCR6 is expressed on the surface of liver sinusoid epithelium cells, while CXCL16 is a characteristic molecule of NKT cells. However, the deficiency of CXCR6 can be compensated for by systemic NKT-cell activation through other methods (181, 188).

Given the significant functions of NKT cells in development of liver tumors, many novel treatments of hepatic cancer based on NKT cells have been invented in recent years. Mice experiments showed that exogenous IL-12 and α-Galcer (186), direct transfer of ex vivo modulated NKT cells (189), tumor antigens (190, 191), and even antigens of some microorganisms (e.g. LPS from bacteria (182) and some recombinant oncolytic viruses (192)are used to potentiate the anti-tumor effect of NKT cells. Low protein diet (193)and blockade of PD-1/PD-L1 axis (194)are also found useful in suppressing hepatic tumors. Notably, clinical trials have confirmed the effectiveness of some NKT-related treatments. For example, in 2021, Tian-Tian Li et al. (195) reported that stereotactic body radiotherapy had positive effects on peripheral NKT cells in HCC patients, which is associated with a higher overall survival. These results indicate NKT cells as a very promising therapeutic target.

## Perspectives and conclusion

In this review, we mainly discussed how NKT cells are activated and the functions of NKT cells during the pathogenesis and development of some liver diseases. It is widely acknowledged that the role NKT cells play in the immunity of the liver and even the whole body is indispensable, and treatment focused on modulating NKT-cell activity is becoming more and more promising. However, considering the dual functions NKT cells have in many liver diseases, the treatment tactics should be studied thoroughly.

In addition, since this review mainly focuses on the physiology of type I NKT cells (iNKT cells), more work needs to be done for a better understanding of type II NKT cells which are more abundant in the human liver than in mice (196). Type II NKT cells are normally considered to be anti-inflammatory, and regulate type I NKT cells (iNKT) and other immune cells and favor tumor growth (101, 197). However, some also report the role that Type II NKT cells play in promoting chronic inflammation (166). Further studies are needed to better demonstrate the functions of Type II NKT cells and how these cells interact with type I NKT cells as well as other participants in our immune system.

Recently, gene analysis has cast new light on NKT researches. Single-cell RNA sequencing indicates distinct populations of functional NKT cell subsets with differences on gene and epigenetic levels (198, 199). This offers a deeper understanding, and is likely to guide future studies within this field.

## Author contributions

XG, QC and XM have equal contributions to this study. XG and HZ designed the whole study. JW and CC conducted the statistical analysis. XG, QC, and XM draft the manuscript. JG, YR, and SW made the relevant edits to the manuscript. XG, QC and XM revised the manuscript. All authors read and approved the final manuscript.

## Funding

This study was supported by grants awarded by the National Science and Technology Major Project of China (NO 2018ZX10302206), Science and Technology Major Projects of Zhejiang Province (NO 2018C04016), and the Science and Technology Major Projects of Ningbo (NO 2016C51008).

## Conflict of interest

The authors declare that the research was conducted in the absence of any commercial or financial relationships that could be construed as a potential conflict of interest.

## Publisher’s note

All claims expressed in this article are solely those of the authors and do not necessarily represent those of their affiliated organizations, or those of the publisher, the editors and the reviewers. Any product that may be evaluated in this article, or claim that may be made by its manufacturer, is not guaranteed or endorsed by the publisher.
